# Preparation of Antioxidant Protein Hydrolysates from *Pleurotus geesteranus* and Their Protective Effects on H_2_O_2_ Oxidative Damaged PC12 Cells

**DOI:** 10.3390/molecules25225408

**Published:** 2020-11-19

**Authors:** Xiyu Liao, Zhenjun Zhu, Shujian Wu, Mengfei Chen, Rui Huang, Juan Wang, Qingping Wu, Yu Ding

**Affiliations:** 1 Department of Food Science and Technology, Institute of Food Safety and Nutrition, College of Science & Engineering, Jinan University, Guangzhou 510632, China; 1834181017lxy@stu2018.jnu.edu.cn (X.L.); zzj1904@jnu.edu.cn (Z.Z.); sjwu@stu2018.jnu.edu.cn (S.W.); pinky7@stu2017.jnu.edu.cn (M.C.); hr123@stu2019.jnu.edu.cn (R.H.); 2 State Key Laboratory of Applied Microbiology Southern China, Guangdong Institute of Microbiology, Guangdong Academy of Sciences, Guangzhou 510070, China; wuqp203@163.com; 3 Guangdong Provincial Key Laboratory of Microbial Safety and Health, Guangdong Institute of Microbiology, Guangdong Academy of Sciences, Guangzhou 510070, China; 4 Guangdong Open Laboratory of Applied Microbiology, Guangdong Institute of Microbiology, Guangdong Academy of Sciences, Guangzhou 510070, China; 5 College of Life Science and Technology, Jinan University, Guangzhou 510632, China; 6 College of Food Science, South China Agricultural University, Guangzhou 510642, China; wangjuan@scau.edu.cn

**Keywords:** *Pleurotus geesteranus*, protein hydrolysate, antioxidant activity, oxidative stress, PC12 cell

## Abstract

*Pleurotus geesteranus* is a promising source of bioactive compounds. However, knowledge of the antioxidant behaviors *of P. geesteranus* protein hydrolysates (PGPHs) is limited. In this study, PGPHs were prepared with papain, alcalase, flavourzyme, pepsin, and pancreatin, respectively. The antioxidant properties and cytoprotective effects against oxidative stress of PGPHs were investigated using different chemical assays and H_2_O_2_ damaged PC12 cells, respectively. The results showed that PGPHs exhibited superior antioxidant activity. Especially, hydrolysate generated by alcalase displayed the strongest 2,2-diphenyl-1-picrylhydrazyl (DPPH) radical scavenging activity (91.62%), 2,2-azino-bis (3-ethylbenzothia zoline-6-sulfonic acid) (ABTS) radical scavenging activity (90.53%), ferric reducing antioxidant power, and metal ion-chelating activity (82.16%). Analysis of amino acid composition revealed that this hydrolysate was rich in hydrophobic, negatively charged, and aromatic amino acids, contributing to its superior antioxidant properties. Additionally, alcalase hydrolysate showed cytoprotective effects on H_2_O_2_-induced oxidative stress in PC12 cells via diminishing intracellular reactive oxygen species (ROS) accumulation by stimulating antioxidant enzyme activities. Taken together, alcalase hydrolysate of *P. geesteranus* protein can be used as beneficial ingredients with antioxidant properties and protective effects against ROS-mediated oxidative stress.

## 1. Introduction

Reactive oxygen species (ROS) such as superoxide anion free radical, hydroxyl free radical, and hydrogen peroxide are constantly generated in living organisms [1]. These free radicals, which are physiologically produced, play important roles in biological systems that exert diverse functions like cell signaling, homeostasis, autophagy, and cell division [2]. However, an excessive amount of ROS will lead to an imbalance of oxidation and antioxidant effects, which trigger an oxidative stress response in vivo [3]. Oxidative stress can result in protein damage, DNA mutation, and oxidation of membrane phospholipids [4], leading to various chronic diseases, such as neurodegenerative disease, chronic inflammatory disease, cardiovascular disease, and cancer [5]. Hence, using antioxidants to resist or alleviate oxidative stress is essential. Compared with natural antioxidants, synthetic antioxidants may have potential risks. Therefore, researchers are working to develop natural antioxidants from food which can be used to prevent oxidation in place of synthetic antioxidant food additives with side effects [6].

Protein hydrolysates (mainly contains antioxidant peptides), derived from natural sources, have gained growing interest as potential natural antioxidants due to their little or no negative side effects, lower molecular weight, easy absorption, high activity, and low cost [7]. Nowadays, antioxidant protein hydrolysates from various sources, including mushroom [8], soy [9], milk [10], *Cyprinus carpio* skin gelatin [11], and duck breast [12], have been investigated. Regarding the protein sources of bioactive peptides, one of the promising foodstuffs are mushrooms [13]. Firstly, the average protein content in mushrooms is about 23.80 ± 9.82 g/100 g dry weight [14], which is higher than most vegetables. Mushrooms are also rich in various bioactive compounds, such as polysaccharides, phenols, peptides, carotenoids, ergosterol, and vitamins C and E [15,16,17]. Additionally, mushrooms grow faster when compared to other plants, which makes them a relatively abundant source of natural bioactive compounds for commercial applications [18]. Thus, mushrooms are widely used for pharmaceutical purposes and as functional foods for centuries [19].

*Pleurotus geesteranus*, commonly known as the oyster mushroom, is one type of edible mushrooms consumed and cultivated worldwide [20]. It has been gaining increasing attention due to the high quality of proteins, richness of dietary fiber, and pleasant umami taste [21]. Nowadays, *P. geesteranus* is considered to have immense potential as a valuable medicinal compound [22]. Different studies have demonstrated that the polysaccharides, polysaccharide-protein, and other nutrients of *P. geesteranus* exhibited anti-tumor [21], hepatoprotective [23], and antioxidant [24] effects. However, the antioxidant properties of protein hydrolysates from *P. geesteranus* have rarely been reported.

Hence, this study was focused on the antioxidant activity of protein hydrolysates from *P. geesteranus*. For this purpose, the proteins of *P. geesteranus* were extracted and hydrolyzed by different commercial proteases (papain, alcalase, flavourzyme, pepsin, and pancreatin). Subsequently, the antioxidant activities of protein hydrolysates from *P. geesteranus* were assessed using the chemical methods as well as H_2_O_2_-damaged PC12 cells. The cytoprotective mechanism in PC12 cells was also investigated.

## 2. Results and Discussion

### 2.1. Enzymatic Hydrolysis

**Figure 1:**
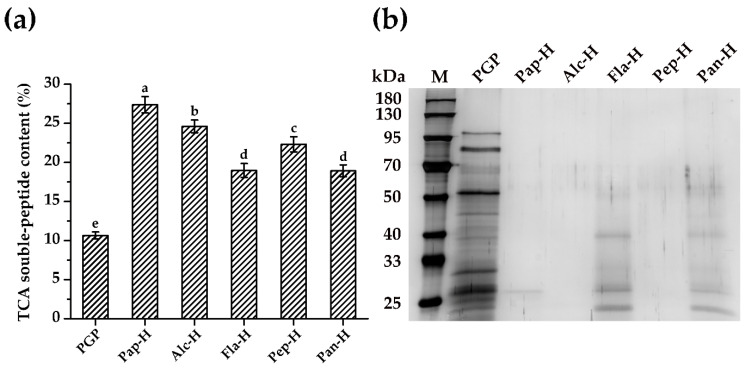
The efficiency of different enzymes in hydrolyzing *Pleurotus geesteranus* protein (PGP). (**a**) TCA-soluble peptide content in PGP and its hydrolysates; Data are expressed as mean ± SD (*n* = 3). Bars with different alphabets indicate statistically significant difference between the means (*p* < 0.05). (**b**) Sodium dodecyl sulfate-polyacrylamide gel electrophoresis (SDS-PAGE) profile of PGP and its hydrolysates generated by various enzymes; 20 μg of protein were loaded into the corresponding lane. M, marker; Pap-H, papain hydrolysate; Alc-H, alcalase hydrolysate; Fla-H, flavourzyme hydrolysate; Pep-H, pepsin hydrolysate; Pan-H, pancreatin hydrolysate.

To verify the effectiveness of protease hydrolysis, the TCA-soluble peptide content in *P. geesteranus* protein (PGP) and *P. geesteranus* protein hydrolysates (PGPHs) was investigated. As shown in Figure 1a, the yield of the TCA-soluble peptide was dramatically increased after being hydrolyzed by the five enzymes for 2 h and papain hydrolysate showed the highest content (27.38%; *p* < 0.05), followed by alcalase hydrolysate (24.61%), pepsin hydrolysate (22.31%), flavourzyme hydrolysate (18.97%), and pancreatin hydrolysate (18.93%), respectively. Furthermore, the molecular weight distribution of polypeptides in PGP and PGPHs were analyzed. As shown in Figure 1b, the non-hydrolyzed PGP mainly consisted of bands ranging from 20–120 kDa. Within this range, there were at least five major protein bands at around 27 kDa, 30 kDa, 55 kDa, 88 kDa, and 115 kDa, respectively. After hydrolysis, the major protein bands of alcalase and pepsin hydrolysates completely disappeared. Meanwhile, the major protein bands of PGP were effectively hydrolyzed by papain, flavourzyme, and pancreatin, except for the protein bands with the molecular weight of 27 kDa and 40 kDa as evidenced by the reduction in their intensity. The difference of TCA-soluble peptide content and protein bands in PGPHs may be associated with the cleavage patterns [25] and specificities of the enzyme used [26]. Overall, the enhanced TCA-soluble peptide content and the diminished protein bands suggested that papain, alcalase, flavourzyme, pepsin, and pancreatin could efficiently hydrolyze PGP.

### 2.2. Antioxidant Activity of PGP and PGPHs

**Figure 2:**
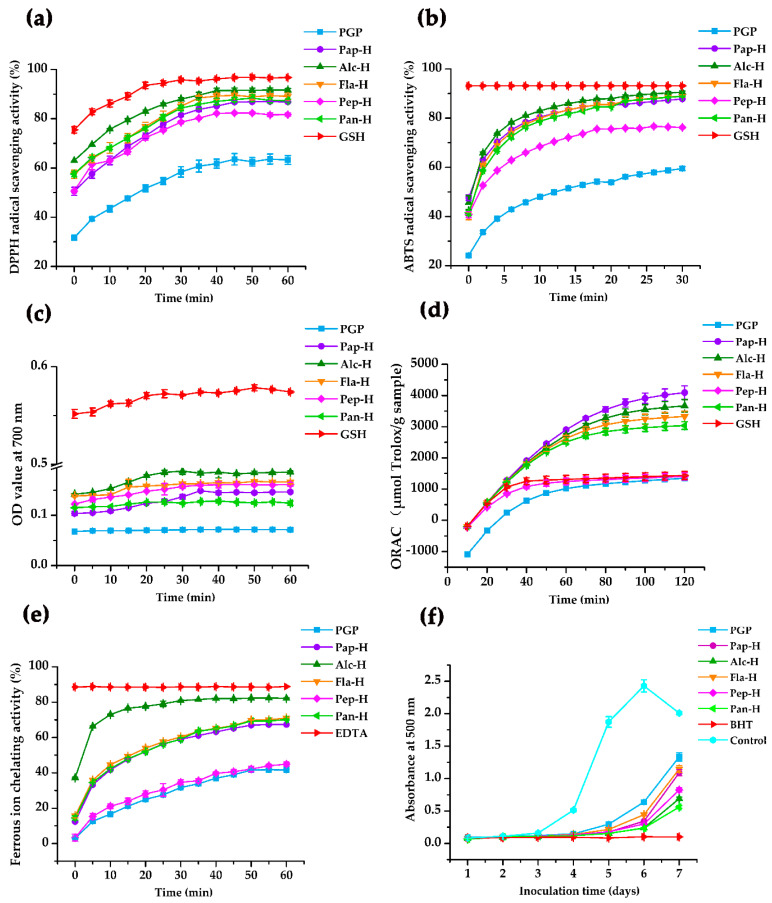
Antioxidant properties of PGP and its hydrolysates. (**a**) DPPH radical scavenging activity; (**b**) ABTS radical scavenging activity; (**c**) Ferric reducing antioxidant power; (**d**) Oxygen radical absorbance capacity (ORAC); (**e**) Ferrous ion chelating activity; (**f**) Inhibition of linoleic acid peroxidation. PGP, *Pleurotus geesteranus* protein; Pap-H, papain hydrolysate; Alc-H, alcalase hydrolysate; Fla-H, flavourzyme hydrolysate; Pep-H, pepsin hydrolysate; Pan-H, pancreatin hydrolysate. Data are expressed as mean ± SD (*n* = 3).

In this study, the antioxidant activity of PGP and PGPHs were analyzed using six different methods: DPPH radical scavenging activity, ABTS radical scavenging activity, ferric reducing antioxidant power (FRAP), oxygen radical absorbance capacity (ORAC), ferrous ion chelating activity, and lipid peroxidation inhibition. As shown in Figure 2, PGP presented a certain antioxidant activity in different antioxidant assays. Moreover, its antioxidant activity changed over time, except for the FRAP. However, it was noteworthy that the hydrolysates presented a significantly higher DPPH radical scavenging activity, ABTS radical scavenging activity, FRAP, ORAC value, ferrous ion chelating activity, and lipid peroxidation inhibition activity than their parental proteins. The possible reason for the enhanced antioxidant performance of PGPHs is that the concentrations of the amino acids with significant antioxidant activity (including Tyr, Trp, Cys, Met, and His) [9,27] in PGPHs were higher than that in PGP (Table 1). Consistent with our results, Farzaneh et al. [13] and Cotabarren et al. [28] also indicated that the hydrolysates exhibited better antioxidant activity than their initial proteins. Collectively, our result together with previous study suggested that enzymatic hydrolysis is an effective method to obtain bioactive compounds from protein sources. Particularly, the DPPH radical scavenging activity of PGPHs (81.66–91.62% at 0.5 mg/mL) was higher than sardinelle protein hydrolysates (70 ± 0.8% at 5 mg/mL) [29], chicken skin gelatin peptides (14.55% at 5 mg/mL) [30], and peptides from corn gluten meal (34.16 ± 0.79% at 0.5 mg/mL) [31]. Meanwhile, PGPHs had a higher ORAC value (1405.56–4090.86 μM TE/g) when compared to milk bioactive peptides (29.8–52.5 μM TE/g) [10] and cod protein hydrolysate (940 μM TE/g) [32], which revealed that PGPHs could be used as valuable ingredients with superior antioxidant properties.

For PGPHs, the antioxidant activity changed over time and a high range of differences in the antioxidant activity was found among different hydrolysates. Among them, the DPPH radical scavenging activity of all hydrolysates increased in a similar trend and reached a steady-state around 40 min. Moreover, alcalase and flavourzyme hydrolysates displayed better DPPH radical scavenging activity (91.62% and 89.08%, respectively). In ABTS radical scavenging activity, the activity of all hydrolysates increased rapidly in the first 6 min and the alcalase hydrolysate showed the strongest ABTS free radical scavenging activity from 2 min to the end of the reaction. Moreover, the ferrous ion chelating activity of each hydrolysate increased sharply in the first five min, and the alcalase hydrolysate was the best one during the reaction period. In addition, the ORAC value of different hydrolysates, except for pepsin hydrolysate, increased sharply in the first 60 min. After 2 h, papain and alcalase hydrolysates showed higher ORAC values (4090.86 μM TE/g and 3667.77 μM TE/g, respectively). In the linoleic acid oxidation system, each hydrolysate showed similar inhibitory activity in the first four days. However, alcalase and pancreatin hydrolysates exhibited better inhibitory effects on the seventh day. Taken together, alcalase hydrolysate appeared to have superior antioxidant ability. Many studies revealed that the difference in the hydrolysates’ antioxidant potentials are partly ascribed to their amino acid composition [33,34]. As shown in Table 1, alcalase hydrolysate was characterized by the highest level of hydrophobic AAs (HAAs, including Ala, Pro, Tyr, Val, Met, Cys, Ile, Leu, and Phe), which can interact with free radical via hydrophobic forces thereby increasing the proximity of the radical to the active functional groups [27]. Meanwhile, alcalase hydrolysate enriched the contents of Glu and Asp, which are negatively charged amino acids (NCAAs). According to He et al. [35], NCAA could confer antioxidant activity to peptides due to the abundance of electrons that can be donated to quench free radicals. In addition, alcalase hydrolysate had significantly higher amounts of aromatic amino acids (AAAs). A previous study has demonstrated that the presence of AAAs could enhance the potency of radical scavenging activity by donating protons to stabilize electron-deficient radicals while retaining their stability through resonance structures [7]. Moreover, alcalase hydrolysate was rich in histidine, which has strong radical scavenging activity due to the presence of an imidazole ring [36]. It is therefore understandable that alcalase hydrolysate exhibited superior antioxidant ability among the PGPHs. Previous studies also reported that the alcalase hydrolysate of *Hericium erinaceus* mushroom [37] and rapeseed protein [38] showed stronger antioxidant activity than other protease hydrolysates. Thus, alcalase hydrolysate was chosen for further investigations.

### 2.3. Effects of Alcalase Hydrolysate on Hydrogen Peroxide (H_2_O_2_)-Induced Oxidative Damage

**Figure 3:**
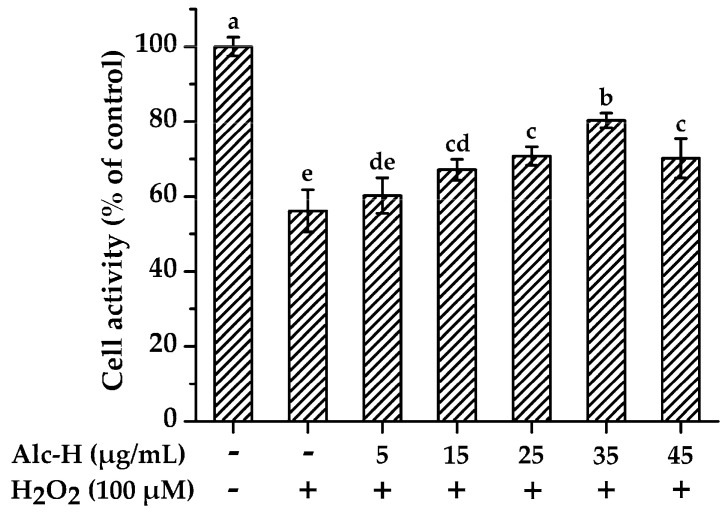
Protective effects of alcalase hydrolysate (Alc-H) on hydrogen peroxide (H_2_O_2_)-induced cytotoxicity. PC12 cells were pretreated with various concentrations of Alc-H (5–45 μg/mL) for 12 h before being exposed to 100 μM H_2_O_2_ for 1 h. Cell viability was determined using the CCK8 assay. Data are expressed as mean ± SD (*n* = 4). Bars with different alphabets indicate statistically significant difference between the means (*p* < 0.05).

To further evaluate the antioxidant behaviors of alcalase hydrolysate in the biological system, we verified the effects of alcalase hydrolysate on H_2_O_2_-damaged PC12 cells. Firstly, the cytotoxicity of alcalase hydrolysate was determined. As shown in Supplementary Figure S1, PC12 cells treated with alcalase hydrolysate (5–45 μg/mL) for 12 h showed no significant change in the cell viability compared with the control group. In contrast, the cell viability was significantly reduced by H_2_O_2_ treatment (Supplementary Figure S2), which was around 50% when using 100 μM H_2_O_2_ treated for 1 h. Therefore, this concentration (IC_50_) was chosen for the following experiments. As shown in Figure 3, alcalase hydrolysate (5–35 μg/mL) attenuated H_2_O_2_-induced cell damage in a dose-dependent manner. Compared to H_2_O_2_ treatment alone, 35 μg/mL of alcalase hydrolysate restored cell viability up to 80.32%. However, at a higher concentration (45 μg/mL), the cell viability was significantly decreased. Similar consequences were found by Saw et al. [39] and Zou et al. [40]. Poljsak et al. [3] indicated that excessive antioxidants may act as pro-oxidants and cause oxidative stress. Therefore, 35 μg/mL of alcalase hydrolysate was used in the subsequent experiments.

### 2.4. Effects of Alcalase Hydrolysate on H_2_O_2_-Induced ROS Accumulation

**Figure 4:**
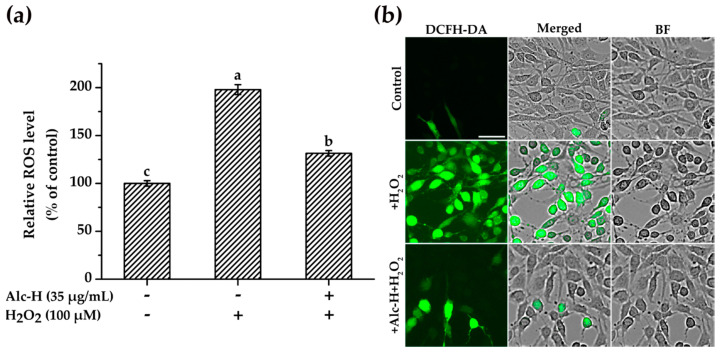
Effects of alcalase hydrolysate (Alc-H) on H_2_O_2_-induced intracellular reactive oxygen species (ROS) accumulation in PC12 cells. (**a**) Fluorescent intensity of ROS inside the cells. PC12 cells were pretreated with Alc-H (35 μg/mL) for 12 h before being exposed to 100 μM H_2_O_2_ for 1 h. Data are expressed as mean ± SD (*n* = 4). Bars with different alphabets indicate statistically significant difference between the means (*p* < 0.05). (**b**) Fluorescent images of ROS inside the cells were captured by Cytation 5 fluorescent cell imager. Cells were pretreated with Alc-H (35 μg/mL) for 12 h before stimulation with 100 μM H_2_O_2_ for 1 h. Bar = 50 μm.

Overproduction of ROS is known to induce oxidative stress [40]. Previous studies have suggested that protein hydrolysates can act as antioxidants, which effectively scavenge ROS [41,42]. Hence, we evaluated whether alcalase hydrolysate affected the levels of ROS in H_2_O_2_-damaged PC12 cells. As shown in Figure 4a, exposure to H_2_O_2_ alone dramatically increased ROS levels when compared to the control (*p* < 0.05), indicating that H_2_O_2_ triggered oxidative stress in PC12 cells through the production and accumulation of ROS. As expected, pretreatment with alcalase hydrolysate significantly decreased ROS levels. Concomitant results were observed in cell fluorescence images (Figure 4b). As seen, the brightest group was observed from the cells treated with H_2_O_2_ alone. However, the green fluorescence was greatly reduced by pretreatment with alcalase hydrolysate (35 μg/mL). In sum, these results provided promising evidence that alcalase hydrolysate played a vital role in diminishing the ROS accumulation to protect PC12 cells against oxidative damage.

### 2.5. Effects of Alcalase Hydrolysate on Cellular Antioxidant Enzyme Activities

**Figure 5:**
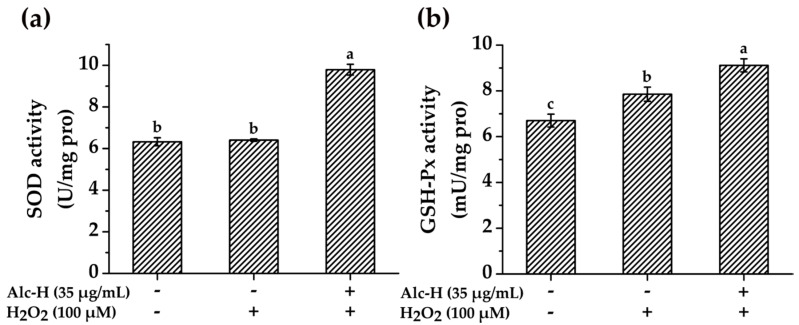
Effects of alcalase hydrolysate (Alc-H) on the activities of antioxidant enzymes in PC12 cells. (**a**) SOD activity; (**b**) GSH-Px activity. PC12 cells were pretreated with Alc-H (35 μg/mL) for 12 h before being exposed to 100 μM H_2_O_2_ for 1 h. Data are expressed as mean ± SD (*n* = 4). Bars with different alphabets indicate statistically significant difference between the means (*p* < 0.05).

Cells have a complex antioxidant defense system, which contains enzymatic and non-enzymatic systems [43], to regulate cellular redox homeostasis to protect against ROS damage. Endogenous antioxidant enzymes, such as SOD and GSH-Px, maintain the oxidative equilibrium in the first line [44]. SOD converts superoxide radicals into hydrogen peroxide, which is then changed into water and oxygen by GSH-Px [45]. To further elucidate the cytoprotective mechanism of alcalase hydrolysate, its impact on the activities of SOD and GSH-Px were investigated. As shown in Figure 5, the SOD and GSH-Px activities of the cells treated by H_2_O_2_ were 6.41 U/mg pro and 7.85 U/mg pro, respectively, which were the same with or higher than the control groups (*p* < 0.05), indicating that the antioxidative defense system was activated when the cells were treated by H_2_O_2_. When the cells were pretreated by alcalase hydrolysate, the activities of SOD and GSH-Px were significantly higher than that of H_2_O_2_-treated group (*p* < 0.05), suggesting that alcalase hydrolysate inhibited ROS accumulation by increasing the activities of SOD and GSH-Px. Comparably, Chai et al. [46] reported that lantern fish (*Benthosema pterotum*) hydrolysates suppressed ROS generation by up-regulating the antioxidant enzyme activities in H_2_O_2_-stimulated SH-SY5Y cells. Overall, alcalase hydrolysate exerted cytoprotective effects in PC12 cells against H_2_O_2_-induced oxidative damage. The protective effect could be partially due to its ability to diminish the intracellular ROS accumulation and enhance antioxidant enzyme activities.

## 3. Materials and Methods

### 3.1. Materials and Reagents

Fresh fruiting bodies of *P. geesteranus* were purchased from the Baiguyuan planting base (Foshan, China). Papain, alcalase, and flavourzyme were purchased from Solarbio Life Sciences (Beijing, China). Pancreatin was bought from Yuanye Bio-Technology (Shanghai, China). Pepsin, l-glutathione reduced, 2,2′-azobis-2-amidinopropane-dihydrochloride (AAPH), fluorescein sodium salt, 1,1-diphenyl-2-picrylhydrazyl (DPPH), linoleic acid, ammonium thiocyanate, and 30% H_2_O_2_ were obtained from Sigma-Aldrich (Shanghai, China). Ferrous chloride was purchased from Macklin Biochemical (Shanghai, China). The broad range molecular weight marker (10–180 kDa) was from Sangon Biotech (Shanghai, China). The rat pheochromocytoma line (PC12) cells were purchased from the Cell Bank of the Institute of Biochemistry and Cell Biology, Chinese Academy of Sciences (Shanghai, China). Dulbecco’s modified Eagle’s medium (DMEM), 0.25% trypsin-EDTA, fetal bovine serum (FBS), and phosphate-buffered saline (PBS, pH 7.4) were obtained from Gibco (Grand Island, NY, USA). Penicillin-Streptomycin was from BBI Life Science (Shanghai, China). BCA protein assay kit, reactive oxygen species (ROS), superoxide dismutase (SOD), and glutathione peroxidases (GSH-Px) assay kits were purchased from Beyotime Biotechnology Institute (Shanghai, China). Cell counting kit-8 (CCK8) was obtained from Bimake (Houston, TX, USA). Other reagents were all in analytical grade.

### 3.2. Preparation of Sample

The fresh fruiting bodies were washed with deionized water and dried at 80 °C. Then the dried mushrooms were crushed into powder and stored at a glass dryer before use.

#### 3.2.1. Extraction of PGP

The protein extraction of *P. geesteranus* was performed according to the procedures described by Qian et al. [47] with some modifications. The mushroom powder was soaked in deionized water (1:15, *w/v*). After extraction for 12 h with stirring at 4 °C, the homogenate was centrifuged at 12,000× *g* for 15 min at 4 °C. Ammonium sulfate was added to the supernatant to obtain 75% relative saturation and the precipitate was allowed to form overnight (12 h) at 4 °C. Following the centrifugation at 12,000× *g* for 20 min at 4 °C, the protein precipitation was resuspended in a small volume of deionized water and dialyzed against deionized water at 4 °C for 24 h. Finally, the PGP was obtained by freeze-drying (FreeZone 4.5, Labconco, MO, USA) and stored at −20 °C before use.

#### 3.2.2. Preparation of PGPHs

The PGP was hydrolyzed by five different proteases (papain, alcalase, flavourzyme, pepsin, and pancreatin) respectively at their optimum hydrolysis conditions (as shown in Table 2). PGP was dissolved in deionized water to make a 2% (*w*/*v*, protein content basis) solution placed on a magnetic stirring water bath. The mixture was incubated at an appropriate temperature and adjusted to the optimal pH of each enzyme. Enzymatic hydrolysis was initiated by the addition of enzyme at an enzyme/substrate ratio of 4% (*w*/*w*, protein basis). The pH of the reaction mixture was maintained by the addition of 1 M NaOH or 1 M HCl. After 2 h of enzymatic hydrolysis, the mixture reaction was boiling for 10 min to terminate the reaction. Then, each hydrolysate was cool to room temperature and centrifugated at 10,000× *g* for 20 min at 4 °C. The supernatants were stored at −20 °C as PGPHs.

### 3.3. TCA-Soluble Peptide Content

TCA-soluble peptide content was determined according to Zhu et al. [48] with some modifications. The PGP and PGPHs were mixed with an equal amount of 20% (*w*/*v)* TCA solution followed by vortex for 10 s and standing for 30 min, and then centrifuged at 10,000× *g* for 5 min. The TCA-soluble peptide content in the supernatant was analyzed using the BCA method [49] with bovine serum albumin (BSA) as the standard. The yield of the TCA-soluble peptide was calculated using the following equation:

TCA − soluble peptide (%) = A/B × 100%,(1)

where A is the peptide content of the supernatant and B is the total protein content of the sample before hydrolysis.

### 3.4. Sodium Dodecyl Sulfate-Polyacrylamide Gel Electrophoresis (SDS-PAGE)

SDS-PAGE analysis of PGP and PGPHs was performed according to Prieto et al. [50]. Electrophoresis was conducted at a constant current of 60 V to move the proteins through the 5% stacking gel, followed by 100 V to resolve the proteins in a 10% separating gel. Then, the gel was stained with silver staining. The image was subsequently captured using Tanon 2500 image analyzer (Shanghai, China).

### 3.5. Amino acid (AA) Composition

To analyze AA composition of PGP and PGPHs, samples were hydrolyzed with 6 M HCl under the nitrogen atmosphere at 110 °C for 22 h. After vacuum evaporation to remove the acid, the hydrolysates were diluted, filtered, and analyzed with an automatic amino acid analyzer (Hitachi L-8900, Tokyo, Japan) according to Liu et al. [51]. For the determination of sulfur-containing AAs (Met and Cys), the samples were oxidized with performic acid before hydrolysis in HCl. To determine tryptophan, alkaline hydrolysis was performed as described by Kimatu et al. [52]. The final results of the AA composition were expressed as mg/100 g protein.

### 3.6. Determination of Antioxidant Activities

#### 3.6.1. DPPH Radical Scavenging Activity

The ability of PGP and PGPHs to scavenge the DPPH radical was measured using the method of Yang et al. [6] with minor modifications. An aliquot of 0.5 mL of PGP and PGPHs (0.5 mg/mL) were mixed with the same volume of DPPH (0.1 mM) in 95% ethanol. The mixture was vortexed for 10 s and incubated in dark at room temperature. Absorbance at 517 nm was recorded every 5 min over 60 min. The DPPH radical scavenging effect was calculated using the following formula:

(2) $$\mathrm{DPPH}\;\mathrm{radical}\;\mathrm{scavenging}\;\mathrm{activity}\;(\%)\;=\;(1-\left(A_{1}-A_{2}\right)/A_{3})\;\times \;100\%,$$

where A_1_ is the absorbance of the mixture of sample and DPPH solution, A_2_ is the absorbance of the sample blended with ethanol, and A_3_ is the absorbance of deionized water mingled with DPPH solution. Glutathione (GSH) was used as the positive control.

#### 3.6.2. ABTS Radical Scavenging Activity

The ABTS radical scavenging activity of the sample was evaluated according to Pimentel et al. [53] with some modifications. Briefly, the ABTS radical was generated by mixing 7 mM ABTS and 2.45 mM potassium persulfate in the same volume, and the mixture was kept in dark at 25 °C for 12–16 h. Before used, the absorbance of the ABTS solution at 734 nm was controlled to be 0.70 ± 0.05 by diluting with phosphate buffer (5 mM, pH 7.4). Then, 10 μL samples (0.5 mg/mL) were mixed with 200 μL ABTS radical solution and incubated at room temperature. Absorbance at 734 nm was recorded every 2 min over 30 min. Deionized water instead of the sample was used as the blank and GSH was used as the positive control. The ABTS radical scavenging activity was calculated using the following formula:

(3) $$\mathrm{ABTS}\;\mathrm{radical}\;\mathrm{scavenging}\;\mathrm{activity}\;(\%)\;=\;(\left(A_{b}-A_{s}\right)/A_{b})\;\times \;100\%,$$

where $A_{b}$ and $A_{s}$ represent absorbance of the blank and sample, respectively.

#### 3.6.3. Ferric Reducing Antioxidant Power (FRAP)

The FRAP assay was performed using a previous method described by Muhammad et al. [54]. In brief, 5 mL of sodium phosphate buffer (0.2 M, pH 6.6) and 5 mL of 1% (*w*/*v*) potassium ferricyanide were added into 2 mL of samples (0.5 mg/mL). The resulting mixture was incubated at 50 °C. At intervals of 5 min for the 60 min, 0.6 mL of sample was added by 0.25 mL of 10% (*w*/*v*) trichloroacetic acid. After centrifugation at 3000× *g* for 10 min, 0.25 mL of supernatant was collected and mixed with 0.25 mL of deionized water and 0.5 mL of 0.1% (*w*/*v*) FeCl_3_. After incubation for 10 min, the absorbance was determined spectrophotometrically at 700 nm.

#### 3.6.4. Oxygen Radical Absorbance Capacity (ORAC)

ORAC assay was carried out according to the procedure reported by Dong et al. [55] with some modifications. All reagents and samples were prepared in phosphate buffer (0.075 M, pH 7.4). Aliquots (20 µL) of samples (0.1 mg/mL), standard (Trolox at a range of concentrations from 0 to 480 µM, R^2^ = 0.9924) or blank (phosphate buffer) were pipetted into a 96-well microplate, mixed with 120 μL fluorescein (80 nM), and pre-incubated in the dark at 37 °C for 20 min. The reaction started by adding 25 μL of 2.2′-azobis-2-methyl-propanimidamide (AAPH) (150 mM) to each well. Fluorescence (excitation: 485nm, emission: 538 nm) was recorded every 2 min over 120 min. The results were expressed as μM Trolox equivalent (TE)/g of a sample.

#### 3.6.5. Ferrous Ion Chelating Activity

The ability of PGP and PGPHs to chelate ferrous ions was measured following the method of Zhang et al. [56] with minor modifications. Samples solutions were prepared in deionized water to a final concentration of 0.5 mg/mL. A sample solution (4 mL of 0.5 mg/mL) was premixed with FeCl_2_ (200 μL of 2 mM) and deionized water (12 mL) in a reaction tube. At intervals of 5 min for the 60 min, ferrozine solution (20 μL of 5 mM) was added in 810 μL of sample and mixed vigorously. Subsequently, the absorbance of the mixture was detected spectrophotometrically at 562 nm after standing for 10 min. Deionized water without samples was used as the blank. The percentage chelating effect was expressed using the following equation:

(4) $$\mathrm{Ferrous}\;\mathrm{ion}\;\mathrm{chelating}\;\mathrm{activity}\;(\%)\;=\;(\left(A_{b}-A_{s}\right)/A_{b})\;\times \;100\%,$$

where $A_{b}$ and $A_{s}$ represent absorbance of the blank and sample, respectively.

#### 3.6.6. Inhibition of Linoleic Acid Peroxidation

The lipid peroxidation inhibition capacity of PGP and PGPHs was measured in a linoleic acid emulsion system according to the method of Zhao et al. [57]. Samples (0.625 mL) were added in 10 mL of 50 mM phosphate buffer (pH 7.0) and then mixed with 0.13 mL of linoleic acid and 10 mL of 99.5% ethanol. The total volume was then adjusted to 25 mL with deionized water to obtain a final 0.1 mg/mL sample concentration. The mixture was incubated in at 40 ± 1 °C for seven days in dark. The degree of oxidation of linoleic acid was evaluated at 24 h intervals by measuring the ferric thiocyanate value. The reaction mixture (100 μL) was mixed with 4.7 mL of 75% ethanol, 0.1 mL of 30% ammonium thiocyanate, and 0.1 mL of 20 mM ferrous chloride solution in 3.5% HCl. After 3 min incubation, the growing color, which represents the linoleic acid oxidation, was measured at 500 nm. BHT and deionized water instead of samples were used as positive and blank controls, respectively.

### 3.7. Effects of Alcalase Hydrolysate on PC12 Cells Proliferation

PC12 cells were cultured in DMEM supplemented with 10% (*v*/*v*) fetal bovine serum, 0.1% (*v*/*v*) penicillin-streptomycin solution, and 5% CO_2_ at 37 °C in incubator. Firstly, cells (1.5 × 10^5^ cells/well) were seeded into a 96-well plate with 100 µL of culture media and incubated 24 h. Then, 100 µL of alcalase hydrolysate (5, 15, 25, 35, and 45 μg/mL, respectively) was added to the sample group and incubated for 12 h. Finally, the cells were incubated with 100 µL of CCK8 solution for 1 h. Absorbance was detected by a microplate reader (Epoch 2, BioTek, Winooski, VT, USA) at 450 nm. DMEM instead of the sample was used as a control. The cell viability was calculated by the following equation:

(5) $$\mathrm{Cell}\;\mathrm{activity}\;(\%)\;=\;A_{s}/A_{c}\;\times \;100\%,$$

where $A_{s}$ and $A_{c}$ are the absorbances of the sample and control, respectively.

### 3.8. Inducing PC12 Cells with H_2_O_2_

PC12 cells (1.5 × 10^5^ cells/well) were seeded into a 96-well plate with 100 µL of culture media and incubated 36 h at 37 °C. Then, 100 µL of H_2_O_2_ (25, 50, 100, 200, and 400 µM) was added into the cells and incubated for 1 h, while 100 µL of DMEM was added into the control group. The optimal H_2_O_2_ concentration was chosen when the cell viability was close to 50%. Cell viability was measured by the CCK8 method as mentioned in 3.7 and calculated by the following equation:

(6) $$\mathrm{Cell}\;\mathrm{activity}\;(\%)\;=\;A_{m}/A_{c}\;\times \;100\%,$$

where $A_{m}$ and $A_{c}$ are the absorbances of the model and control, respectively.

### 3.9. Cytoprotective Effects of Alcalase Hydrolysate on H_2_O_2_-Damaged PC12 Cells

PC12 cells (1.5 × 10^5^ cells/well) were seeded into a 96-well plate and incubated in 100 µL of culture media for 24 h at 37 °C. Then, 100 µL of alcalase hydrolysate (5, 15, 25, 35, and 45 μg/mL, respectively) was added into the sample group. DMEM instead of alcalase hydrolysate was used as the model and control groups. After 12 h, 100 µL of H_2_O_2_ (100 µM) was added into the model and sample groups, and incubated for 1 h at 37 °C, respectively. Further, 100 µL of DMEM instead of H_2_O_2_ was added into the control group. Finally, cell viability was measured by the CCK8 method.

### 3.10. Determination of Intracellular ROS Accumulation

Details of cell treatments with alcalase hydrolysate (35 μg/mL) and H_2_O_2_ (100 μM) were the same as 3.9. Measurement of intracellular ROS was assessed according to a previously reported method [58] with some modifications. First, 2,7-dichlorodihydrofluorescein diacetate (DCFH-DA) (100 µL, 10 μM) was seeded into cells for 30 min at 37 °C. After 30 min, the solution was removed and the cells were washed with DMEM to remove extracellular probes. Subsequently, fluorescence intensity was measured with excitation and emission wavelengths of 488 nm and 525 nm, and cell fluorescence images were captured using a Fluorescent Cell Imager (Cytation 5, Biotek, USA).

### 3.11. Measurement of SOD and GSH-Px Levels

PC12 cells were seeded onto a cell culture flask (100 mm diameter; Corning Inc., Corning, NY, USA) at a density of 3.2 × 10^5^ cells/cm^2^ for 24 h. Firstly, 10 mL of alcalase hydrolysate (35 μg/mL) were added into the sample group and cultured for 12 h. Then, 10 mL of alcalase hydrolysate solution replaced by DMEM were used for the model and control groups. After 12 h, 10 mL of H_2_O_2_ (100 mM) was added into the model and sample groups and incubated for 1 h at 37 °C. Ten mililiter of DMEM instead of H_2_O_2_ was added into the control group. Subsequently, the cells were collected and lysed on ice with cell lysis buffer for 30 min. After treatment, intracellular SOD and GSH-Px levels were evaluated using the commercial kits (Beyotime Institute of Biotechnology, Shanghai, China) according to the manufacturer’s instructions. Protein content was quantified using a BCA protein assay kit (Beyotime Institute of Biotechnology, Shanghai, China) with bovine serum albumin (BSA) as the standard.

### 3.12. Statistical Analysis

All experiments were performed in triplicate (*n* = 3) and the results were expressed as means ± standard deviations (SDs). ANOVA test using SPSS 17.0 (SPSS Inc., Chicago, IL, USA) was employed to analyze the experimental data. Differences among the means were compared using Duncan’s multiple range test. Differences were considered significant at *p* < 0.05.

## 4. Conclusions

In sum, the present study demonstrated that *P. geesteranus* and its hydrolysates are promising sources of antioxidant compounds. Among different hydrolysates, the alcalase one exhibited superior in vitro antioxidant activity due to the richness of HAAs, NCAAs, and AAAs within the hydrolysates. Meanwhile, alcalase hydrolysate exerted antioxidant activities against oxidative stress by down-regulating ROS accumulation and up-regulating antioxidant enzyme activities in H_2_O_2_-damaged PC12 cells. These results suggested that the potential of antioxidant protein hydrolysates, derived from *P. geesteranus* through alcalase hydrolysis, could serve as a source of bioactive molecules in alleviating oxidative stress.

## Figures and Tables

**Table 1:** Amino acid composition of PGP and its hydrolysates (mg/100 g).

Amino Acids	PGP ^1^	Pap-H ^2^	Alc-H ^3^	Fla-H ^4^	Pep-H ^5^	Pan-H ^6^
Asp	35.53 ± 0.13	71.57 ± 0.43	109.26 ± 0.89	86.25 ± 0.24	63.28 ± 0.25	87.73 ± 0.92
Thr	20.34 ± 0.11	40.25 ± 0.25	62.17 ± 0.26	48.67 ± 0.09	35.11 ± 0.16	48.50 ± 0.57
Ser	16.51 ± 0.05	33.30 ± 0.21	51.97 ± 0.16	40.30 ± 0.07	29.17 ± 0.11	40.90 ± 0.42
Glu	29.80 ± 0.17	54.87 ± 0.25	86.45 ± 0.53	65.75 ± 0.12	49.95 ± 0.17	68.68 ± 0.56
Gly	13.67 ± 0.04	27.95 ± 0.10	45.55 ± 0.20	34.29 ± 0.04	25.02 ± 0.05	35.09 ± 0.25
Ala	16.45 ± 0.10	31.48 ± 0.06	58.22 ± 0.19	38.52 ± 0.04	28.17 ± 0.06	39.05 ± 0.30
Cys	4.38 ± 0.09 ^f^	7.06 ± 0.16 ^d^	9.92 ± 0.34 ^a^	8.17 ± 0.10 ^c^	5.22 ± 0.08 ^e^	8.75 ± 0.39 ^b^
Val	16.50 ± 0.02	34.75 ± 0.19	58.94 ± 0.30	41.82 ± 0.05	29.68 ± 0.14	42.86 ± 0.38
Met	3.07 ± 0.12 ^f^	8.78 ± 0.09 ^d^	14.32 ± 0.09 ^a^	10.67 ± 0.05 ^c^	3.78 ± 0.17 ^e^	11.21 ± 0.24 ^b^
Ile	12.21 ± 0.04	27.68 ± 0.09	46.56 ± 0.21	33.54 ± 0.08	22.98 ± 0.15	34.48 ± 0.42
Leu	16.80 ± 0.10	37.99 ± 0.04	63.83 ± 0.33	46.27 ± 0.16	33.40 ± 0.17	47.88 ± 0.28
Tyr	16.05 ± 0.17 ^e^	34.77 ± 0.25 ^c^	44.88 ± 0.15 ^a^	40.94 ± 0.09 ^b^	27.74 ± 0.28 ^d^	41.07 ± 0.76 ^b^
Phe	17.15 ± 0.21	34.91 ± 0.11	53.67 ± 0.56	41.23 ± 0.14	29.89 ± 0.18	42.08 ± 0.35
Lys	19.95 ± 0.21	30.39 ± 0.15	47.28 ± 0.66	35.16 ± 0.10	26.62 ± 0.30	38.47 ± 0.24
His	8.51 ± 0.24 ^f^	15.13 ± 0.04 ^d^	23.28 ± 0.45 ^a^	17.42 ± 0.06 ^c^	13.13 ± 0.16 ^e^	18.72 ± 0.08 ^b^
Arg	9.85 ± 0.19	4.93 ± 0.10	20.57 ± 0.11	6.09 ± 0.06	10.96 ± 0.13	8.03 ± 0.06
Pro	12.29 ± 0.11	29.54 ± 1.28	43.13 ± 0.34	34.94 ± 0.13	25.69 ± 0.10	35.71 ± 1.60
Trp	4.16 ± 0.11 ^f^	14.85 ± 0.15 ^b^	13.20 ± 0.13 ^d^	13.49 ± 0.12 ^c^	9.36 ± 0.08 ^e^	16.69 ± 0.16 ^a^
HAA ^7^	119.05 ± 1.05 ^f^	261.81 ± 2.42 ^d^	406.68 ± 2.65 ^a^	309.60 ± 0.95 ^c^	215.91 ± 1.38 ^e^	319.80 ± 4.87 ^b^
NCAA ^8^	65.34 ± 0.30 ^f^	126.44 ± 0.68 ^d^	195.71 ± 1.43 ^a^	151.99 ± 0.36 ^c^	113.24 ± 0.36 ^e^	156.41 ± 1.47 ^b^
AAA ^9^	37.36 ± 0.49 ^f^	84.54 ± 0.51 ^d^	111.76 ± 0.84 ^a^	95.66 ± 0.35 ^c^	66.99 ± 0.53 ^e^	99.85 ± 1.27 ^b^

^1^*Pleurotus geesteranus* protein, ^2^ Papain hydrolysate, ^3^ Alcalase hydrolysate, ^4^ Flavourzyme hydrolysate, ^5^ Pepsin hydrolysate, ^6^ Pancreatin hydrolysate, ^7^ Hydrophobic amino acids including Ala, Pro, Tyr, Val, Met, Cys, Ile, Leu, and Phe, ^8^ Negatively charged amino acids including Glu and Asp, ^9^ Aromatic amino acids including Tyr, Phe, and Trp. ^a–f^ Data are expressed as mean ± SD (*n* = 3). Different alphabets indicate statistically significant difference between the means (*p* < 0.05).

**Table 2:** Hydrolysis conditions of various enzymes.

	Papain	Alcalase	Flavourzyme	Pepsin	Pancreatin
Temperature (°C)	55	50	50	37	37
pH	7	9	7	2	7.5
Time (h)	2	2	2	2	2
E ^1^/S ^2^ ration (*w/w*)	4/100	4/100	4/100	4/100	4/100
Substrate concentration (*w/v*)	2/100	2/100	2/100	2/100	2/100

^1^ Enzyme; ^2^ Substrate.

## Supplementary Materials

The following are available online, Figure S1: Viability of PC12 cells after treatment with various doses of alcalase hydrolysate (Alc-H). Figure S2: Viability of PC12 cells after treatment with different concentrations of hydrogen peroxide (H_2_O_2_).
