# Supplementary material for: Preparation of Antioxidant Protein Hydrolysates from Pleurotus geesteranus and Their Protective Effects on H2O2 Oxidative Damaged PC12 Cells
Source: Molecules. 2020 Nov 19;25(22):5408. doi: 10.3390/molecules25225408 (PMC7699252; doi:10.3390/molecules25225408)
Supplement: Supplementary file 1 [file molecules-25-05408-s001.pdf]

Supplementary Materials for

Preparation of Antioxidant Protein Hydrolysates from *Pleurotus geesteranus* and Their Protective Effects on H_2O_2 Oxidative Damaged PC12 Cells

Xiyu Liao [1,2], Zhenjun Zhu [1], Shujian Wu [1,2], Mengfei Chen [1,2], Rui Huang [1,2], Juan Wang [3], Qingping Wu [2] and Yu Ding [1,2,*]

[1] Department of Food Science and Technology, Institute of Food Safety and Nutrition, College of Science & Engineering, College of Life Science and Technology, Jinan University, Guangzhou 510632, China; 1834181017lxy@stu2018.jnu.edu.cn (X.L.); zzj1904@jnu.edu.cn (Z.Z.); sjwu@stu2018.jnu.edu.cn (S.W.); pinky7@stu2017.jnu.edu.cn (M.C.); hr123@stu2019.jnu.edu.cn (R.H.)

[2] Guangdong Institute of Microbiology, Guangdong Academy of Sciences, State Key Laboratory of Applied Microbiology Southern China, Guangdong Provincial Key Laboratory of Microbial Safety and Health, Guangdong Open Laboratory of Applied Microbiology, Guangzhou 510070, China; wuqp203@163.com

[3] College of Food Science, South China Agricultural University, Guangzhou 510642 China; wangjuan@scau.edu.cn

* Correspondence: dingyu@jnu.edu.cn; Tel.: +86-20-85222379

Academic editor: Angela Cardinali

Received: 22 October 2020; Accepted: 16 November 2020; Published: date

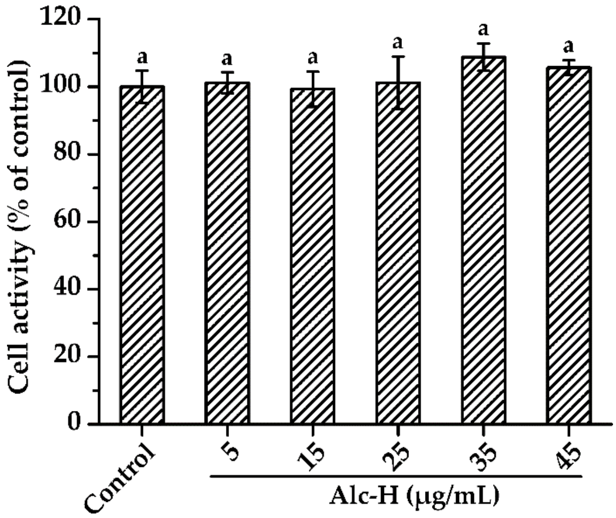

Figure S1. Viability of PC12 cells after treatment with various doses of alcalase hydrolysate (Alc-H). PC12 cells were treated for 12 h with the noted concentrations of Alc-H. Cell viability was determined using CCK8 assay. Data are expressed as mean ± SD (n = 4). Bars with different alphabets indicate statistically significant difference between the means ($p < 0.05$).

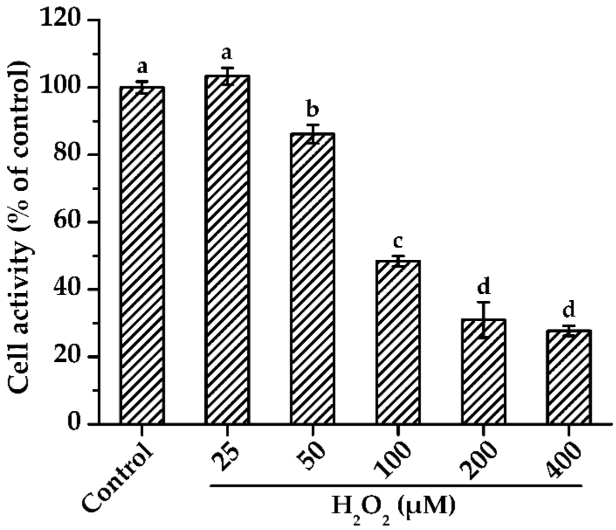

Figure S2. Viability of PC12 cells after treatment with different concentrations of hydrogen peroxide (H_2O_2). PC12 cells were treated with the noted concentrations of H_2O_2 for 1 h. Cell viability was determined using CCK8 assay. Data are expressed as mean ± SD ($n = 4$). Bars with different alphabets indicate statistically significant difference between the means ($p < 0.05$).
